# Complete mitochondrial genome of the Antarctic cod icefish, *Pagothenia borchgrevinki* (Perciformes: Nototheniidae)

**DOI:** 10.1080/23802359.2016.1180554

**Published:** 2016-07-06

**Authors:** Yimeng Liu, Min Yang, Tao Zhou, Hu Xing, Liangbiao Chen, Dongsheng Zhang

**Affiliations:** Key Laboratory of Aquacultural Resources and Utilization, Ministry of Education, College of Fisheries and Life Sciences, Shanghai Ocean University, Shanghai, P.R. China

**Keywords:** Antarctic, bald notothen, mitogenome

## Abstract

Antarctic icefish *Pagothenia borchgrevinki* is an ideal model for studying heat stress mechanisms. The complete mitochondrial genome of *P. borchgrevinki* was sequenced in this study. The genome sequence is 17,299 bp in length, which comprises 13 protein-coding genes, 22 tRNAs, 2 rRNAs and a control region. The overall base composition is 20.45% G, 25.11% A, 29.46% T and 24.98 C%, with an A:T content of 54.57%.

The Antarctic cod icefish *Pagothenia borchgrevinki* (Perciformes: Nototheniidae), also called Bald Notothen, live under the sea ice around Antarctica. It has developed many adaptations to the extremely cold environment, including antifreeze glycoproteins that prevent ice expansion in its body fluid (O'Grady et al. 1982; Praebel et al. 2009). Although the bald notothen is highly cold-adapted and stenothermal, it is capable of some degree of tolerance to acute heat challenge if pre-treated with warm acclimation (Bilyk & Devries 2011). As this fish is cold resistant and less sensitive to environmental variation, it is a good model for biological research on thermal adaptation. Thermal adaptation is highly related to energy metabolism, where the 13 mitochondrially encoded genes play key roles. The complete mitochondrial genome sequence will be useful for understanding the evolution and adaptation of Antarctic Notothenioids under extreme environment. In this study, the complete mitochondrial genome of *P. borchgrevinki* was determined. Fish sample was collected from Prydz Bay near Chinese Zhongshan Station, Antarctica (69°22’S, 76°22’E). The species is stored at Key Laboratory of Aquacultural Resources and Utilization, Shanghai Ocean University, with accession number ANT005. The sample DNA is available upon request.

The complete mitogenome of *P. borchgrevinki* (Genbank accession number: KU951144) was 17,299 bp in length, with overall base composition of 20.45% G, 25.11% A, 29.46% T and 24.98 C%. The circular genome has 37 conserved genes, contains 13 protein-coding genes, 22 tRNA genes, 2 rRNA genes and 1 control region (CR or D-loop). Most of its mitochondrial genes appear to be encoded on the heavy strand except for NADH dehydrogenase subunit 6 (ND6) and eight tRNA genes (tRNA-Gln, Ala, Asn, Cys, Tyr, Ser, Pro and Glu), which were encoded on the light strand. In 13 protein-coding genes, the shortest one was ATP8 gene (168 bp) and the longest one was ND5 gene (1839 bp). Twelve of 13 protein coding genes started with a common initiation codon ATG, while COI utilized GTG. Ten of 13 protein-coding genes ended with complete (TAA) or incomplete (TA) or incomplete (T) stop codons. ND6 stopped with AGG. ATPase8 and ND5 both ended with TAG. The 22tRNA genes ranged in size from 65 bp in tRNA-Cys to 75 bp in tRNA-Leu and tRNA-Lys. The 12S and 16S rRNA genes were 947 bp and 1689 bp, respectively. D-loop was 1211 bp in length.

To investigate the phylogenetic relationship among Antarctic Notothenioids, we downloaded the mitochondrial genome sequences of eight currently available Notothenioids, including *Chionodraco myersi* (accession no. NC_010689), *Chaenocephalus aceratus* (NC_015654), *Champsocephalus gunnari* (NC_018340), *Pleuragramma antarctica* (NC_015652), *Parachaenichthys charcoti* (NC_026578), *Notothenia coriiceps* (NC_015653) and *Dissostichus eleginoides* (NC_018135), *Chionodraco hamatus* (KT921282), together with African lungfish *Protopterus annectens* (NC_018822) as an outgroup species. The concatenated nucleotide sequences of 13 protein-coding genes, 22 tRNAs, 2 rRNAs and the D-loop of 10 species were aligned with the CLUSTALW program (Larkin et al. 2007) and refined by eye. Phylogenetic trees were reconstructed with the concatenated nucleotide alignment using MEGA6 (Tamura et al. 2013) for neighbour-joining, maximum likelihood and maximum parsimony methods. Tree topology was evaluated by 1000 bootstrap replicates. Different methods give the same tree topology, and the result indicates that *P. borchgrevinki* mitogenome is close to that of *P.charcoti* (Figure 1).

**Figure 1.**
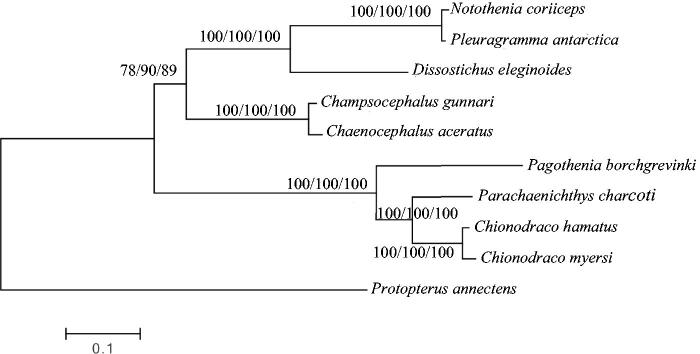
Phylogenetic tree of Antarctic Notothenioids, with African lungfish *P. annectens* as an outgroup. The topology of phylogenetic tree was inferred from neighbour-joining, maximum likelihood and maximum parsimony methods. Bootstrap supports for each analysis are indicated at the nodes.
